# Case report: Coexistence of triple-seronegative myasthenia gravis and pathology-proven cryptogenic organizing pneumonia

**DOI:** 10.3389/fneur.2023.1295374

**Published:** 2023-11-16

**Authors:** Shi-Qi Huang, Bin Wang, Lin Gao, Meng Wang, Hong-Dong Zhao, Jian-Quan Shi

**Affiliations:** ^1^Department of Neurology, Nanjing First Hospital, Nanjing Medical University, Nanjing, Jiangsu, China; ^2^Department of Pathology, Nanjing First Hospital, Nanjing Medical University, Nanjing, Jiangsu, China; ^3^Department of Respiratory Medicine, Nanjing First Hospital, Nanjing Medical University, Nanjing, Jiangsu, China

**Keywords:** myasthenia gravis, cryptogenic organizing pneumonia, coexistence, seronegative, antibody

## Abstract

**Objective:**

Emerging evidence shows that patients with myasthenia gravis (MG) were at a higher risk for the co-occurrence of other autoimmune diseases, which reflects phenotypic heterogeneity in MG. The coexistence of MG and cryptogenic organizing pneumonia (COP) has rarely been reported. The present case is to report the coexistence of triple-seronegative MG and pathology-proven COP in a patient.

**Methods:**

The clinical data of the patient were derived from medical records of Nanjing First Hospital, Nanjing Medical University, China. Written informed consent was obtained from the patient.

**Results:**

We presented a 56-year-old man with acute respiratory syndrome, who was diagnosed with COP based on the intra-alveolar fibroinflammatory buds (Masson's bodies) in the pathology of bronchoscopy biopsy. Oral prednisone induced dramatic symptomatic improvement and complete resolution of previous lung lesions. After a stable course of no respiratory symptom for 2 months, he was referred to the neurology department with complaints of fluctuating generalized muscle weakness. He was diagnosed with triple-seronegative MG based on fluctuating weakness, neostigmine test-positivity and RNS-positivity. After three-month treatment with pyridostigmine in combination with tacrolimus, the symptoms gradually improved and he achieved minimal symptom expression.

**Conclusions:**

This case highlights the rare coexistence of triple-seronegative MG and pathology-proven COP. However, a causal association between COP and MG cannot be explicitly ascertained. In future, more data are needed to clarify the relationship, taking into account the limited number of cases reported with this coexistence of the diseases.

## Introduction

Myasthenia gravis (MG) is a rare autoimmune disease, which is characterized by fatigable muscle weakness. The disease is an organ-specific disease mediated by autoantibodies, including antibodies against acetylcholine receptor (AChR), muscle-specific tyrosine kinase (MuSK) and lipoprotein receptor-related protein 4 (LRP4) (1). However, about 10% of MG patients without detectable antibodies are classified as triple-seronegative MG, which represents a heterogeneous group (2). Patients with MG carried an elevated risk of developing other autoimmune diseases simultaneously or successively, which reflects phenotypic heterogeneity in MG (3, 4). As we previously reported, MG could coexsit with autoimmune thyroid disease, Sjögren's syndrome, rheumatoid arthritis, psoriasis, vitiligo, autoimmune hemolytic anemia, idiopathic thrombocytopenic purpura, polymyositis and autoimmune hepatitis (5).

Here, we reported the rare coexistence of triple-seronegative MG and pathology-proven cryptogenic organizing pneumonia (COP) in a patient. COP has no recognizable cause, which is classified as a subtype of idiopathic interstitial pneumonia. To the best of our knowledge, the coexistence of MG and COP in a single patient has not been reported in the literature.

## Case presentation

**Figure 1:**
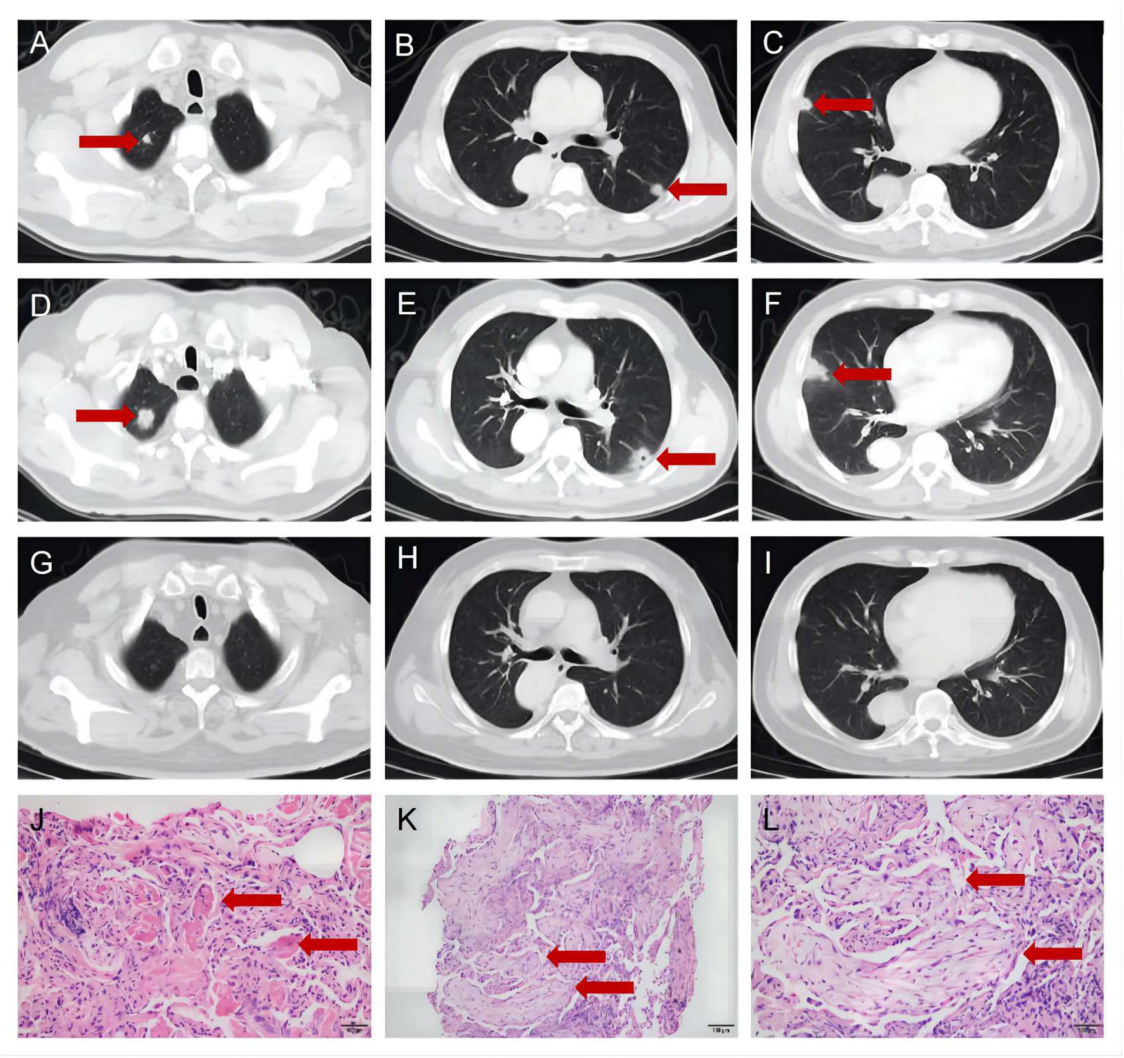
The chest high-resolution computed tomography and pathology of bronchoscopy biopsy. **(A–C)** The chest high-resolution computed tomography at onset indicated irregular nodular opacities in the upper and lower lobe of the right lung and the middle lobe of left lung (Red arrows). **(D–F)** The chest high-resolution computed tomography after 1-week antibiotic therapy indicated expanded lesions (Red arrows). **(G–I)** The chest high-resolution computed tomography at 2-month follow-up showed complete resolution of previous lung lesions (Red arrows). **(J–L)** The pathology of bronchoscopy biopsy showed intra-alveolar fibroinflammatory buds **(**Red arrows, **J)**, in the form of Masson's bodies **(**Red arrows, **K, L)**.

**Figure 2:**
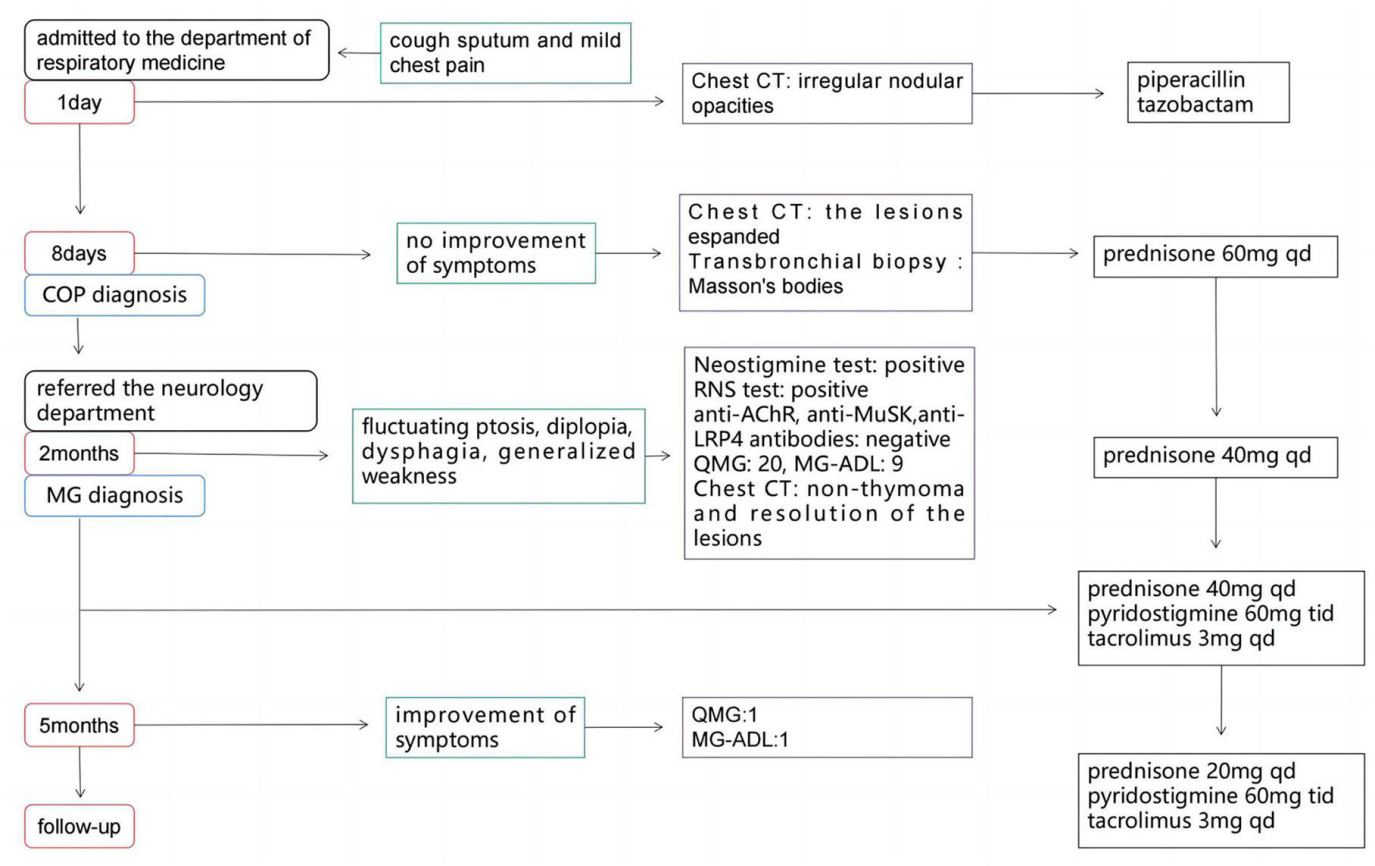
The timeline of the case.

A 56-year-old man presented to the department of respiratory medicine with cough, sputum and mild chest pain for 4 days. He also reported fever with the highest temperature of 38°C. He denied dyspnea, night sweat and weight loss. The patient's medical history include hypertension, which was well controlled. His family history was unremarkable. Lung auscultation revealed normal breath sounds. Routine blood tests and biochemical tests were within normal ranges. Tumor markers and autoantibodies of connective tissue diseases were normal. The elevated C-reactive protein level of 87.27 mg/L (normal range <10 mg/L) was revealed. Subsequent arterial blood gas analysis was normal. The blood culture and sputum culture were negative. High-resolution computed tomography (HRCT) indicated irregular nodular opacitie in the upper lobe and lower lobe of the right lung and the middle lobe of left lung (Figures 1A–C). Initially, he was diagnosed with community-acquired pneumonia and treated with piperacillin/tazobactam. However, the symptoms were not alleviated after 1-week antibiotic therapy. The lesions on chest HRCT expanded gradually (Figures 1D–F). Bronchoalveolar lavage revealed a nonspecific inflammatory pattern with lack of eosinophil and tumor cell. The pathology of bronchoscopy biopsy showed preservation of the lung architecture with a patchy distribution of intra-alveolar fibroinflammatory buds, in the form of Masson's bodies (Figures 1J–L). Based on the findings, lung cancer, infection, connective tissue diseases or other diseases that can cause interstitial pneumonia were ruled out. Hence, he was diagnosed with COP. Oral prednisone (60 mg QD, 0.75 mg/kg of weight) as a monotherapy was initiated with dramatic symptomatic improvement after 1 week. This initial dose was given for 4 weeks and the symptom of COP was remitted completely during the period. Then, the dose of prednisone was reduced by 10 mg/d every 2 weeks. After a stable course of no symptom for 2 months (the dose of prednisone at that time was 40 mg QD), he was referred to the neurology department with complaints of fluctuating ptosis, diplopia, mild dysphagia to solid foods, and generalized muscular weakness for 2 weeks. On neurological examination, he presented bilateral ptosis. He had difficulty in crouching down or walking on his toes or heels. Stretch reflexes, muscle tone, tropism, and sensation were normal. There was bilaterally moderate weakness (Medical Research Council grade 3/5) in shoulder abduction, elbow flexion, wrist extension, hip flexion, knee extension, and foot dorsiflexion. The quantitative MG (QMG) score was 20. The MG activities of daily living (MG-ADL) score was 9. The neostigmine test was positive. Repetitive Nerve Stimulation (RNS) at the deltoid muscle showed a 26.5% decrement. The chest HRCT showed no evidence of thymoma and complete resolution of previous lung lesions (Figures 1G–I). Furthermore, anti-AChR, anti-MuSK and anti-LRP4 antibodies were negative by ELISA. He was diagnosed with triple-seronegative MG based on fluctuating weakness, neostigmine test-positivity and RNS-positivity. In order to decrease the dose of prednisone and to avoid the side effects, pyridostigmine 60 mg TID and tacrolimus 3 mg QD was added (the dose of prednisone at that time was 40 mg QD). After 3-month follow-up (time from the diagnosis of MG), his symptoms gradually improved. The QMG score was 1 (ptosis). The MG-ADL score was 1 (ptosis). Prednisone was tapered to 20 mg QD, while the dose of tacrolimus remained unchanged. Figure 2 summarizes the clinical course.

## Discussion

This report presented the coexistence of triple-seronegative MG and pathology-proven COP in a patient. MG with no detectable AChR, MuSK, or LRP4 antibodies in serum represents a heterogeneous group, which is classified triple-seronegative MG. Different methods are available for the detection of anti-AChR, anti-MuSK or anti-LRP4 antibodies, but the current gold standard remains the radioimmunoprecipitation assay (RIPA) (1). Damato et al. (6) reported that cell-based assay (CBA) can detect the antibodies in a proportion of triple-seronegative MG patients by RIPA. Initially, ELISA was employed to detect serum antibodies, although its sensitivity was relatively low. Recently, we have detected the antibodies in stored serum sample (stored at first visit to the neurology department) by CBA. The anti-AChR, anti-MuSK and anti-LRP4 antibodies were still negative by CBA. Given that the patient was taking prednisone (40 mg QD, for the treatment of COP) when the onset of myasthenic symptom, it is plausible that the immunosuppressive therapy before antibody testing might complicate the identification of the patient's seropositivity. Similar cases have been reported in other antoimmune diseases, such as Celiac disease (7). In addition to detection methods and immunosuppressants in use, other factors might predispose to seronegativity in MG, including unknown autoantibodies, rapid autoantibody clearance and so on (8).

The diagnosis and treatment are more challenging in triple-seronegative MG. As no detectable specific antibodies, diagnosis of triple-seronegative MG should be supported by adequate clinical characteristics and electrophysiological support, especially when the effectiveness of standard treatment is dissatisfactory. From a clinical point of view, it is observed that the clinical characteristics of triple-seronegative MG patients varied among different age groups (9). The overall prognosis of triple-seronegative MG is relatively good, with a low occurrence of crisis. Pyridostigmine is the drug of choice for symptomatic treatment. To improve efficacy and minimize side effects of steroids, a combination of immunosuppressive drugs for the majority of MG patients is recommended, which is based on the sum of guidelines, clinical experience, and relevant research. Efgartigimod is a novel neonatal Fc receptor (FcRn)-inhibiting agent, which have shown significant efficacy in the treatment of anti-AChR generalized MG (10). Efgartigimod is believed to be a new treatment choice for anti-AChR generalized MG and can reduce the dosage of steroids or other immunosuppressants (11). Whether efgartigimod can improve the symptoms of triple-seronegative MG needs further study. Triple-seronegative MG has shown positive response to steroids and pyridostigmine, with approximately a third of patients achieving either complete stable remission or pharmacological remission (9). After 3-month treatment with pyridostigmine in combination with immunosuppressive therapy, the present patient achieved minimal symptom expression, which was defined as MG-ADL score of 0 or 1 and regarded as an practical tool for MG treatment goals (12).

Individuals with MG have an increased vulnerability to other autoimmune diseases, which may manifest before or after the onset of MG. Notably, the incidence of autoimmune thyroid disease appears to be higher in patients with MG (3). We reported a case of triple-seronegative MG with COP. Apart from COP, no other autoimmune diseases were observed during follow-up.

As an idiopathic form of interstitial pneumonia, COP is pathologically characterized by intra-alveolar fibroinflammatory buds (Masson's bodies). The pathogenesis of COP still remains elusive. However, the abnormal immune process might play a pivotal role in the pathogenesis. The diagnosis of COP is an exclusive one to rule out infective pneumonia, eosinophilic pneumonia, other forms of interstitial pneumonia, alveolar hemorrhage, and malignancy and so on (13). The diagnosis of COP requires multidisciplinary approaches, including clinical, radiologic and pathologic expertise. This patient presented with flu-like symptoms prior to the diagnosis of MG, which was initially treated as community-acquired pneumonia. But he had a poor response to antibiotic treatment. Typical radiologic findings, bronchoalveolar lavage and transbronchial biopsy are valuable in the diagnosis of COP. Poletti et al. reported a series of 37 patients with suspected COP. The sensitivity and specificity of bronchoalveolar lavage was 63 and 57%, respectively. Meanwhile, the sensitivity and specificity of transbronchial biopsy was 64 and 86%, respectively (14).

In addition, COP is associated with a high recovery rate if managed appropriately (15). COP patients have a significant response to corticosteroids, which is considered to be one of the strong evidences for the diagnosis of COP. Furthermore, a small proportion of COP patients may recover spontaneously without medical intervention. Corticosteroid therapy is considered the preferred treatment for patients with nonresolving or progressive COP (13). Complete resolution of radiologic infiltrates can be observed after a 3-month period of treatment. However, a retrospective study showed that 31.5% (23/73) of patients relapsed after reduction or cessation of corticosteroids (16). Another study revealed that the recurrence rate after 1-year follow-up was 38.2% (13/30) (17). Clarithromycin has also been proposed for the treatment of COP, with fewer side effects and recurrences than corticosteroids (18). However, it is currently unclear whether macrolides play a specific role in the treatment of COP.

In a retrospective cohort with 91 patients initially diagnosed with COP, 4 patients were eventually identified as OP that was secondary to connective tissue diseases (16). Notably, no clinical or pathological evidence of connective tissue disease was present in the initial evaluation. In other words, secondary OP was the initial manifestation of connective tissue diseases. Hence, it should be acknowledged that OP may not always be cryptogenic. Furthermore, regular prospective monitoring of other clinical manifestation and dynamic assessment of serum biomarkers might be of great significance in identifying potential etiology. During the follow-up period, clinicians need to be aware of screening for other immune-mediated diseases.

More interestingly, OP can coexist with autoimmune neurological diseases. A Japanese retrospective cohort study suggested that 4 in 52 patients with neuromyelitis optica spectrum disease showed pulmonary involvement with a diagnosis of OP, which preceded or coincided with neuromyelitis optica spectrum disease (19, 20). Anti-AQP4 antibody induced complement-dependent cytotoxicity of lung epithelial cells is believed to be partially involved in the development of OP. To our knowledge, there are no reports of the coexistence of COP and triple-seronegative MG in a single patient. Herein, a rare case of triple-seronegative MG in pathologically-confirmed COP is reported. We speculated that the rare co-occurrence might be ascribed to the common immune-mediated mechanisms.

Few reports focus on any association between these two distinct conditions. It could be argued that an immune response to pulmonary antigens might elicit an immune attack against neuromuscular junction. To explore this association is crucial for both diagnosis and management of patients presenting with these rare conditions. There is another possibility that the coexistence of triple-seronegative MG and COP in this case could just be a simple coincidence.

This case highlights the rare coexistence of pathology-proven COP and triple-seronegative MG. However, this descriptive study cannot definitively ascertained the causal association between COP and MG. In future, more data are needed to clarify the relationship, taking into account the limited number of cases reported with this coexistence of the diseases.

## Data availability statement

The original contributions presented in the study are included in the article/supplementary material, further inquiries can be directed to the corresponding author.

## Ethics statement

The studies involving humans were approved by Nanjing First Hospital, Nanjing Medical University. The studies were conducted in accordance with the local legislation and institutional requirements. The participants provided their written informed consent to participate in this study. Written informed consent was obtained from the individual(s) for the publication of any potentially identifiable images or data included in this article.

## Author contributions

SQH: Conceptualization, Data curation, Writing—original draft. BW: Conceptualization, Data curation, Writing—review & editing. LG: Conceptualization, Writing—review & editing. MW: Conceptualization, Data curation, Writing—review & editing. HDZ: Writing—review & editing. JQS: Writing—review & editing.
